# Prevalence and Characteristics of Malaria and Influenza Co-Infection in Febrile Patients: A Systematic Review and Meta-Analysis

**DOI:** 10.3390/tropicalmed7080168

**Published:** 2022-08-05

**Authors:** Polrat Wilairatana, Wanida Mala, Kwuntida Uthaisar Kotepui, Manas Kotepui

**Affiliations:** 1 Department of Clinical Tropical Medicine, Faculty of Tropical Medicine, Mahidol University, Bangkok 10400, Thailand; 2 Medical Technology, School of Allied Health Sciences, Walailak University, Tha Sala, Nakhon Si Thammarat 80160, Thailand

**Keywords:** malaria, *Plasmodium*, influenza, co-infection

## Abstract

Malaria and influenza are co-endemic in several geographical areas, and differentiation of their clinical features is difficult. The present study aimed to qualitatively and quantitatively analyze the prevalence and characteristics of malaria and influenza co-infection in febrile patients. The systematic review was registered at PROSPERO (CRD42021264525). Relevant literature that reported malaria and influenza co-infection in febrile patients were searched in PubMed, Web of Science, and Scopus from 20 June to 27 June 2021 and the risk of bias for each study was assessed. Quantitative analysis included pooled prevalence, and the odds of malaria and influenza virus co-infection among febrile patients were estimated using a random-effects model. Subgroup analyses were performed to summarize the effect estimate for each group. Funnel plot, Egger’s test, and contour-enhanced funnel plot were used to demonstrate any publication bias among outcomes of included studies. Among 4253 studies retrieved, 10 studies that enrolled 22,066 febrile patients with 650 co-infected patients were included for qualitative and quantitative syntheses. The pooled prevalence of malaria and influenza virus co-infection among febrile patients was 31.0% in Nigeria, 1.0% in Tanzania, 1.0% in Uganda, 1.0% in Malawi, 1.0% in Ghana, 0% in Cambodia, 7.0% in the Central African Republic, and 7.0% in Kenya. Meta-analysis also showed co-infection occurrence by chance (*p* = 0.097, odds ratio 0.54, 95% CI 0.26–1.12, *I*^2^ 94.9%). The prevalence of malaria and influenza virus co-infection among febrile patients was heterogeneous by country, characteristics of febrile participants, and diagnostic tests for influenza virus. Further studies should investigate severe clinical manifestations or differentiate clinical outcomes between mono-infected or co-infected individuals, whether the co-infection leads to severe disease outcome.

## 1. Background

Malaria remains a major cause of death in children younger than 5 years old who live in Africa According to the World Health Organization (WHO), more than 241 million malaria cases and 558,000 deaths were reported, almost all (95%) in African countries, while 2% of malaria cases were reported in the WHO South-East Asia Region, and the remaining 3% from other regions [1]. The major cause of malaria in Africa was *Plasmodium falciparum*, although *P. vivax* malaria was also reported, albeit this is less endemic than *P. falciparum* owing to Duffy-negative populations in Africa. Nevertheless, recent evidence suggested that *P. vivax* can infect Duffy-negative individuals [2,3,4], and substantial epidemiological evidence suggests *P. vivax* as a cause of severe malaria [5,6,7]. In contrast to *P. falciparum* and *P. vivax*, a small number of patients develop severe complications derived from *Plasmodium* mixed infection [8], *Plasmodium ovale* [9], or *Plasmodium malariae* [10].

Influenza is an infectious respiratory disease caused by the influenza virus. In humans, severe disease and seasonal epidemics are mostly caused by influenza viruses A and B [11,12]. Previous studies reported the high incidence of influenza in Africa in Nigeria, Tanzania, Kenya, the Central African Republic, and Malawi [11,13,14]. At least 3 million severe influenza cases have been reported, with 290,000 to 650,000 deaths annually [15]. Transmission can occur by direct contact with aerosols and droplets through coughing and sneezing. The clinical symptoms of influenza range from mild respiratory tract infection to acute/chronic disease and are similar to those of other acute febrile illnesses (AFIs), such as pneumonia, typhoid fever, and malaria [11,14]. These overlapping symptoms include fever, chills, headache, and joint and muscle pain [13,16,17]. Children younger than 5 years old are the most vulnerable to morbidity and mortality caused by infection from malaria and influenza [14]. Pregnant women also constitute a risk group for complications from influenza, caused by pregnancy-specific immune changes arising from physiological and anatomical alterations, which lead to high morbidity and mortality especially in the second and third trimesters [11].

As malaria and influenza are co-endemic in several geographical areas, it is difficult to differentiate by the clinical features of the two diseases and other AFIs. To the best of our knowledge, few data regarding the prevalence and characteristics of their co-infection have been published. Therefore, the present study aimed to qualitatively and quantitatively analyze the prevalence and characteristics of malaria and influenza co-infection in febrile patients that have been reported in the literature. The results of this study should guide further investigations of febrile patients by clinicians in co-endemic areas.

## 2. Methods

### 2.1. Protocol and Registration

The systematic review was registered at PROSPERO with the registration number CRD42021264525. Reports of systematic reviews followed the Preferred Reporting Items for Systematic Reviews and Meta-Analyses (PRISMA) statement [18].

### 2.2. Search Strategy

Search terms were constructed and checked with Medical Subject Heading (MeSH). The potentially relevant search terms were combined as “(malaria OR plasmodium OR Paludism OR “Marsh Fever” OR “Remittent Fever”) AND (influenza OR flu OR Influenzas OR Grippe)” (Table S1). The searches were performed in PubMed, Web of Science, and Scopus from 20 June to 27 June 2021 with restriction to the English language but with no restriction on year of publication.

We restricted the literatures in the English language because the articles in English language provided more flexibility for study selection and data extraction by review authors.

An additional search of reference lists of the included studies and another source, Google Scholar, was also performed to assure that potentially relevant studies were not overlooked during the searches.

### 2.3. Eligibility Criteria

The eligibility criteria followed the Participants/Outcome of interest/Context (PICo) principle. P represented febrile patients, I represented co-infection of malaria and influenza virus, and Co-represented the worldwide distribution of co-infection.

Therefore, the inclusion criteria of this study were prospective or retrospective observational studies that reported the concurrent infection of malaria and influenza virus among febrile participants who were suspected of malaria or other flu-like illnesses. The exclusion criteria were non-English articles, studies that reported co-infections but data of co-infections could not be extracted, case reports, case series, letters to editors, reviews, and systematic reviews.

### 2.4. Study Selection and Data Extraction

Potentially relevant studies were selected independently by two authors (M.K. and W.M.) on the basis of eligibility criteria. Disagreements between the two authors were consensualized by another author (P.W.). The flow of study selection after studies were retrieved from databases was as follows: (1) duplicates were removed; (2) title and abstract were screened and unrelated studies were excluded; (3) full texts of studies were examined and unrelated studies were excluded with reasons given; and (4) studies that met the eligibility criteria were included for syntheses. The following data were extracted: first author, publication year, study site, study design, participants and their characteristics, numbers of co-infections, numbers of malaria cases, numbers of influenza cases, diagnostic tests for malaria, and diagnostic tests for influenza. All information was extracted into a pilot standardized datasheet before further analysis. The data extraction was performed by two authors (M.K. and W.M.), and cross-checked by another author (P.W.).

### 2.5. Risk of Bias

The risk of bias for each study was assessed using the Joanna Briggs Institute (JBI) Critical Appraisal Tools for cross-sectional study [19]. The tool assessed the risk of bias for the following eight criteria: explanation of criteria for inclusion of participants, description of subjects and the setting, measurement of exposure validity and reliability, standard criteria used for measurement of the diseases, identification of confounding factors and identification of strategy to deal with them, measurement of outcome validity and reliability, and appropriateness of statistical analysis. A total score of 8 was given to a study that met all eight criteria. Studies with scores of 7–8 indicated a low risk of bias, scores of 5–6 indicated a moderate risk of bias, and scores of less than 5 indicated a high risk of bias.

### 2.6. Data Syntheses

Data syntheses comprise qualitative and quantitative syntheses. Qualitative synthesis is the narrative explanation of data from the included studies, while quantitative synthesis is the statistical analysis of the pooled evidence. The quantitative analysis included: (1) the pooled prevalence of malaria and influenza co-infection among febrile patients; (2) the pooled prevalence of influenza virus among patients with malaria; and (3) the odds of malaria and influenza virus co-infection among febrile patients. The effect estimates and 95% confidence interval (CI) including the pooled prevalence and the pooled odds were estimated using a random-effects model (DerSimonian and Laird). The point estimates and 95% CI of each study for one outcome were visualized in a forest plot. Subgroup analyses of participants, countries, and diagnostic tests for influenza virus were performed to summarize the pooled prevalence per group. Funnel plot, Egger’s test, and contour-enhanced funnel plot were used to demonstrate any publication bias among the outcomes of the included studies. All analyses were performed using Stata version 14 (StataCorp, College Station, TX, USA).

## 3. Results

### 3.1. Search Results

**Figure 1:**
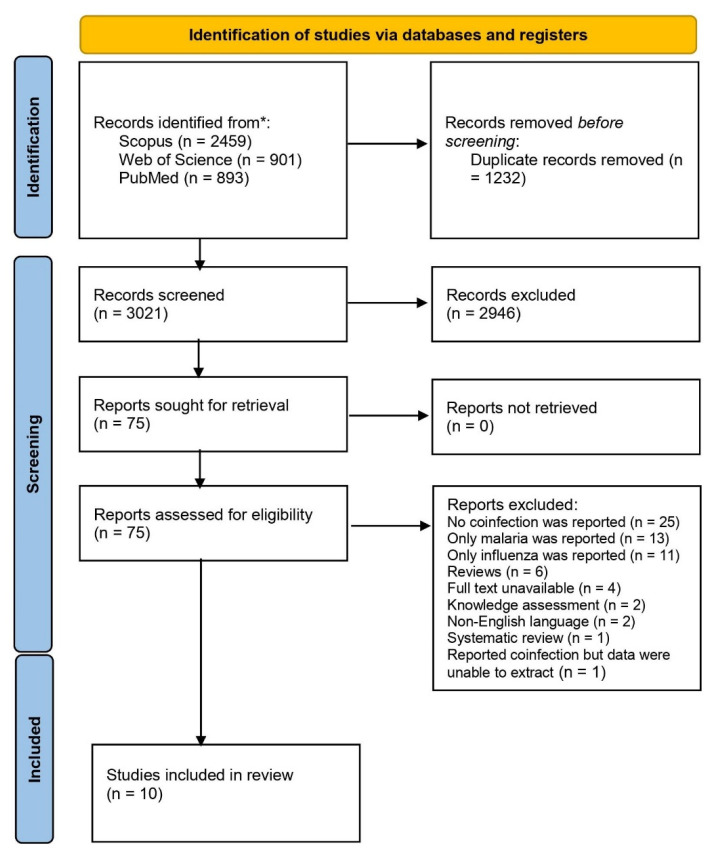
The PRISMA flow diagram showing study selection process. * mean from bibliographical databases.

Among 4253 studies that were retrieved from three databases (2459 from Scopus, 901 from Web of Science, and 893 from PubMed), 1232 duplicates were removed and 3021 studies were screened for titles and abstracts. Next, 2946 unrelated studies were removed, and 75 studies were examined for full texts. Sixty-five studies were then excluded with reasons as follows: (i) 25 in which no co-infection was reported, (ii) 13 in which only malaria was reported, (iii) 11 in which only influenza was reported, (iv) 6 reviews, (v) 4 with full text unavailable, (vi) 2 knowledge assessments, (vii) 2 with non-English language, (viii) 1 systematic review, and (ix) 1 co-infection study from which data could not be extracted. Additional searches from reference lists of the included studies and Google Scholar found no other relevant studies. Therefore, ultimately, 10 studies [11,13,14,20,21,22,23,24,25,26] were included for qualitative and quantitative syntheses (Figure 1).

### 3.2. Characteristics of the Included Studies

**Figure 2:**
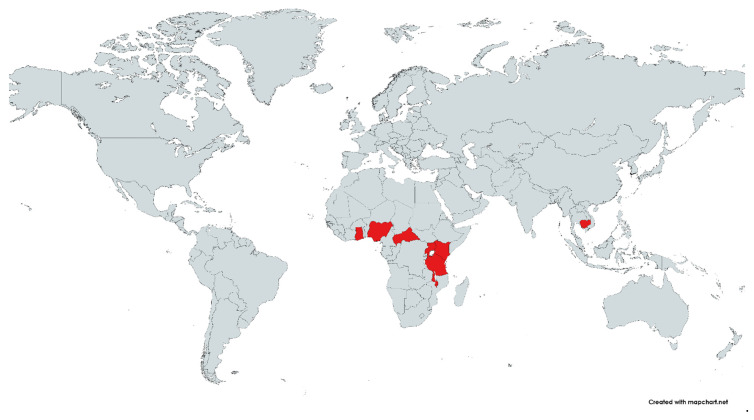
Malaria and influenza co-infection were documented in the following regions (marked in red). The map was made with the help of a map template found at https://mapchart.net/. By referencing mapchart.net, authors are permitted to use, alter, and modify any map made with it.

Characteristics of the included studies are shown in Table 1. All studies were conducted in the period 2006–2018 and published between 2012–2021. Eight studies were conducted in African countries, namely Nigeria [11], Tanzania [20,23], Uganda [21], Malawi [22], Ghana [24], the Central African Republic [13], and Kenya [14]. Two studies [25,26] were conducted in Cambodia (Figure 2). Eight studies [11,13,14,20,23,24,25,26] were observational, while two [21,22] were cohort studies. Two studies enrolled pregnant women [11,22], while other studies [14,20,24] enrolled febrile children, adults [21], and patients in all age groups [13,23,25,26]. For malaria diagnosis, five studies [14,20,22,24,25] used microscopy alone, two studies [13,21] used a rapid diagnostic test (RDT), one study [23] used RDT/microscopy, one study [26] used RDT/polymerase chain reaction (PCR), and one study [11] did not specify the diagnostic method. For influenza diagnosis, six studies [14,21,23,24,25,26] used PCR alone, one study [11] used enzyme-linked immunosorbent assay (ELISA) immunoglobulin M (IgM) alone, one study [20] used ELISA IgM/IgG/PCR, and two studies [13,22] did not specify the diagnostic method (Table 1).

### 3.3. Risk of Bias

The risk of bias for each study was assessed using the JBI Critical Appraisal Tools for cross-sectional study. Six studies [14,20,23,24,25,26] were assessed as having a low risk of bias, while four studies [11,13,21,22] were assessed as having a moderate risk of bias (Table S2).

### 3.4. Pooled Prevalence of Malaria and Influenza Virus Co-Infection

**Figure 3:**
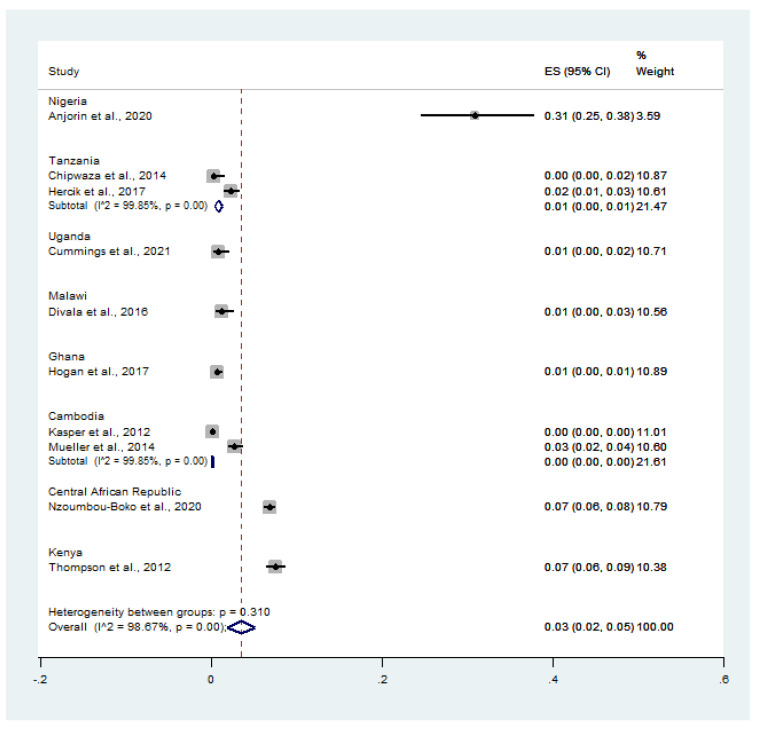
Countries with the highest rates of malaria and influenza co-infection among feverish patients. Percentage weight, each study’s contribution to the pooled effect; black dot on black horizontal line, each study’s point estimate; CI, confidence interval; white diamond, pooled prevalence; ES, effect size (prevalence).

The pooled prevalence of malaria and influenza virus co-infection (650 cases) among febrile patients (22,066 cases) was estimated using data from 10 studies [11,13,14,20,21,22,23,24,25,26]. On stratifying the prevalence by country, the pooled prevalence of malaria and influenza virus co-infection among febrile patients was 31.0% in Nigeria (95% CI: 25.0–38.0%), 1.0% in Tanzania (95% CI: 0–1.0%, *I*^2^: 99.9%), 1.0% in Uganda (95% CI: 0–2.0%), 1.0% in Malawi (95% CI: 0–3.0%), 1.0% in Ghana (95% CI: 0–1.0%), 0% in Cambodia (*I*^2^: 99.9%), 7.0% in the Central African Republic (95% CI: 6.0–8.0%), and 7.0% in Kenya (95% CI: 6.0–9.0%) (Figure 3).

**Figure 4:**
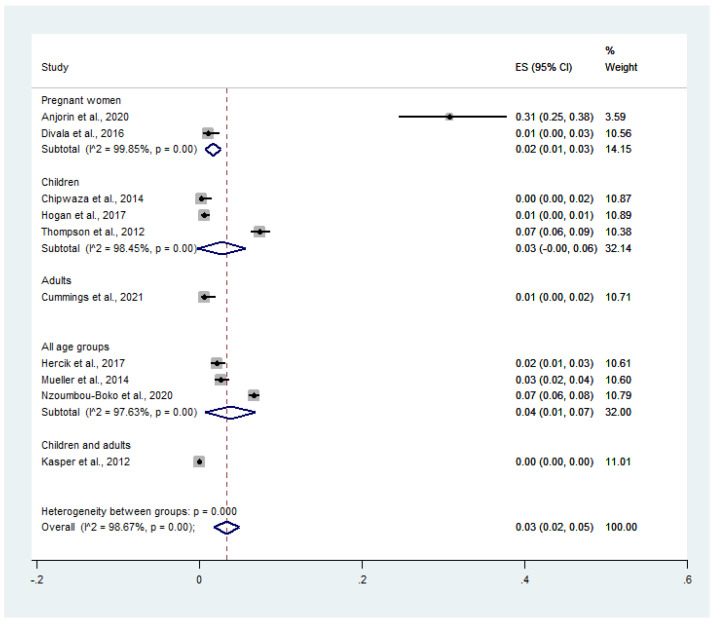
Malaria and influenza co-infection rates in febrile patients, broken down by participant groups. Percentage weight, the fraction of each study’s impact on the pooled effect; black dot on black horizontal line, each study’s point estimate; CI, confidence interval; white diamond, pooled prevalence; ES, effect size (prevalence).

For stratifying the prevalence by groups of participants, the pooled prevalence of malaria and influenza virus co-infection among febrile patients was 2.0% in pregnant women (95% CI: 1.0–3.0%, *I*^2^: 99.9%), 3.0% in children (95% CI: 0–6.0%, *I*^2^: 98.5%), 1.0% in adults (95% CI: 0–2.0%), and 4.0% in all age groups (95% CI: 1.0–7.0%, *I*^2^: 97.6%) (Figure 4).

**Figure 5:**
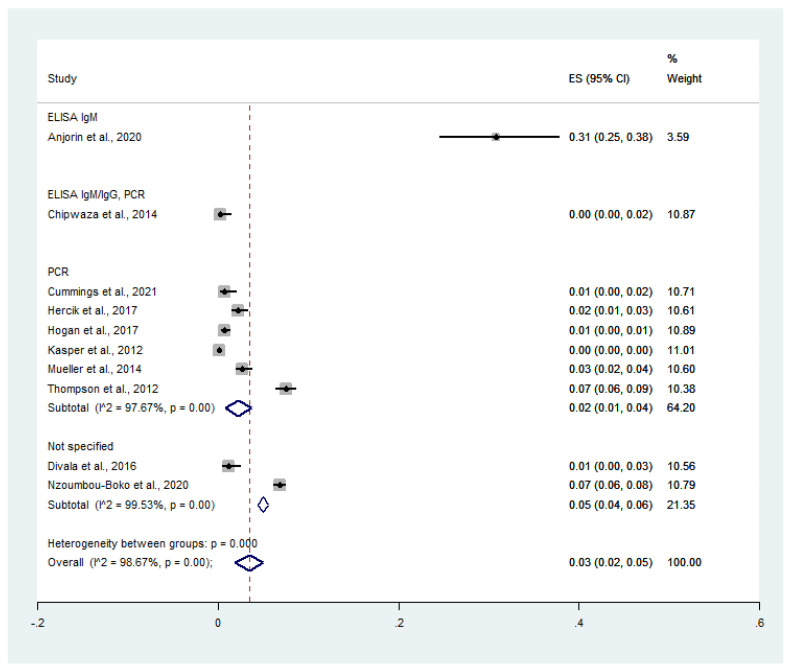
Diagnostic testing for influenza virus reveals the prevalence of malaria and influenza co-infection among feverish patients. Percentage weight, the fraction of each study’s impact on the aggregated effect; black dot on black horizontal line, each study’s point estimate; white diamond, pooled prevalence; CI, confidence interval; ES, effect size (prevalence).

For stratifying the prevalence by diagnostic tests for influenza virus, the pooled prevalence of malaria and influenza virus co-infection among febrile patients was 31.0% in the study using ELISA IgM (95% CI: 25.0–38.0%), 0% in studies using ELISA IgM/IgG/PCR (95% CI: 0–2.0%), 2.0% in studies using PCR (95% CI: 1.0–4.0%, *I*^2^: 97.7%), and 5.0% in studies that did not specify the diagnostic method for influenza virus (95% CI: 4.0–6.0%, *I*^2^: 99.5%). Overall, the pooled prevalence of malaria and influenza virus co-infection among febrile patients was 3.0% (95% CI: 2.0–5.0%, *I*^2^: 98.7%) (Figure 5).

### 3.5. Pooled Prevalence of Influenza Virus Infection among Malaria-Positive Patients

**Figure 6:**
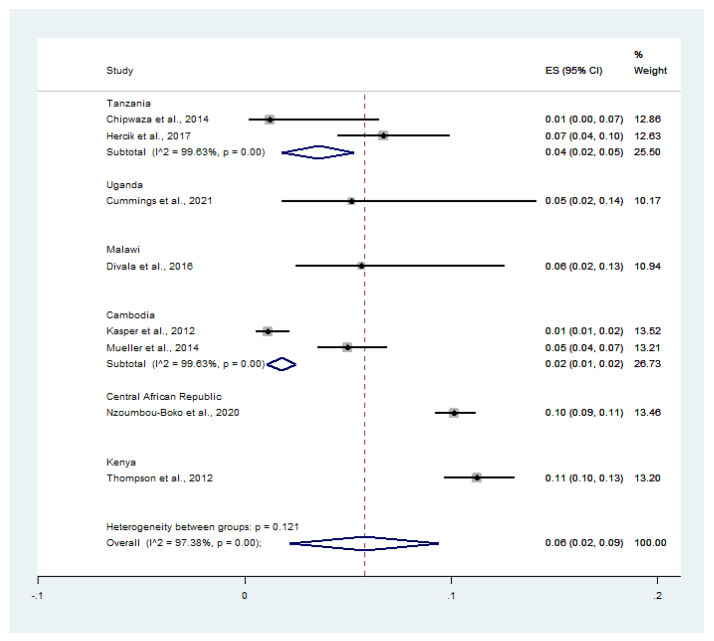
Countries with the highest rates of malaria and influenza co-infection among feverish patients. Percentage weight, the fraction of each study’s impact on the pooled outcome; black dot on black horizontal line, each study’s point estimate; white diamond, pooled prevalence; CI, confidence interval; ES, effect size (prevalence).

The pooled prevalence of influenza virus infection (587 cases) among malaria-positive patients (6847 cases) was estimated using data from eight studies [13,14,20,21,22,23,25,26]. On stratifying the prevalence by country, the pooled prevalence of influenza virus infection among malaria-positive patients was 4.0% in Tanzania (95% CI: 2.0–5.0%, *I*^2^: 99.6%), 5.0% in Uganda (95% CI: 2.0–14.0%), 6.0% in Malawi (95% CI: 2.0–13.0%), 2.0% in Cambodia (95% CI: 1.0–2.0%, *I*^2^: 99.6%), 10.0% in the Central African Republic (95% CI: 9.0–11.0%), and 11.0% in Kenya (95% CI: 10.0–13.0%) (Figure 6).

**Figure 7:**
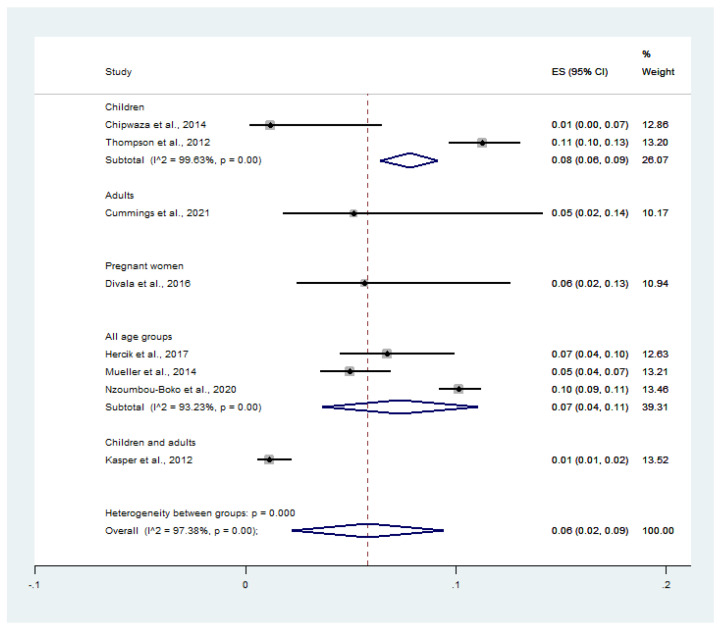
Influenza virus infection rates among malaria patients, broken down by participant groups. Percentage weight, the fraction of each study’s impact on the pooled outcome; black dot on black horizontal line, each study’s point estimate; white diamond, pooled prevalence; CI, confidence interval; ES, effect size (prevalence).

On stratifying the prevalence by groups of participants, the pooled prevalence of influenza virus infection among malaria-positive patients was 8.0% in children (95% CI: 6.0–9.0%, *I*^2^: 99.6%), 5.0% in adults (95% CI: 2.0–14.0%), 6.0% in pregnant women (95% CI: 2.0–13.0%), and 7.0% in all age groups (95% CI: 4.0–11.0%, *I*^2^: 93.2%) (Figure 7).

**Figure 8:**
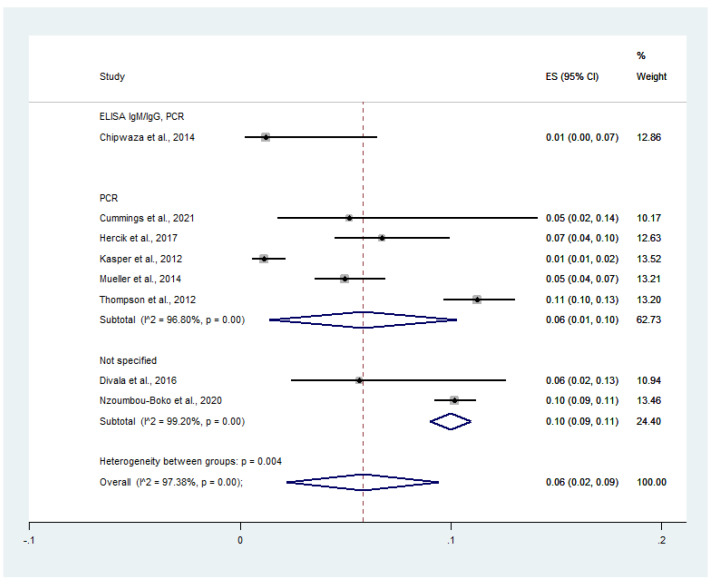
Diagnostic assays for influenza virus infection reveal the prevalence of influenza virus infection among malaria patients. Percentage weight, the fraction of each study’s impact on the pooled outcome; black dot on black horizontal line, each study’s point estimate; white diamond, pooled prevalence; CI, confidence interval; ES, effect size (prevalence).

On stratifying the prevalence by diagnostic tests for influenza virus, the pooled prevalence of influenza virus infection among malaria patients was 1.0% in studies using ELISA IgM/IgG/PCR (95% CI: 0–7.0%), 6.0% in studies using PCR (95% CI: 1.0–10.0%, *I*^2^: 96.8%), and 10.0% in studies that did not specify the diagnostic method for influenza virus (95% CI: 9.0–11.0%, *I*^2^: 99.2%). Overall, the pooled prevalence of influenza virus infection among malaria patients was 6.0% (95% CI: 2.0–9.0%, *I*^2^: 97.4%) (Figure 8).

### 3.6. Odds of Co-Infection

**Figure 9:**
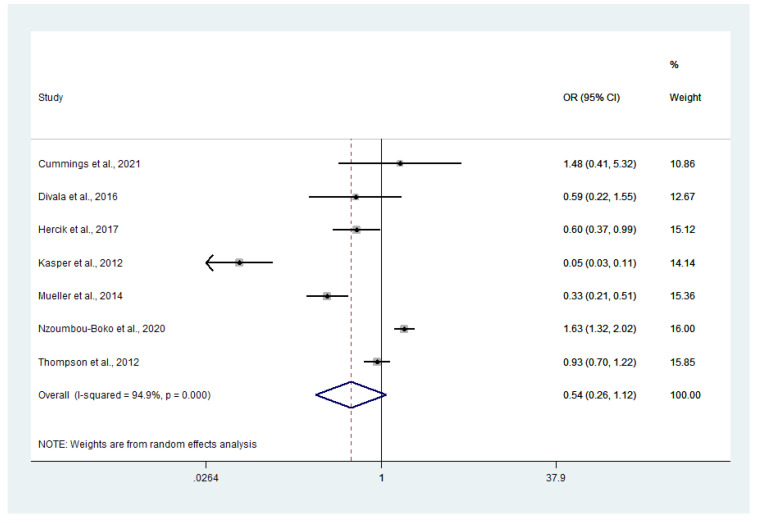
Malaria and influenza co-infections have a high chance of occurring. Percentage weight: each study’s contribution to the pooled effect; black dot on black horizontal line, each study’s point estimate.

Odds of malaria and influenza virus co-infection were estimated using the data from seven studies [13,14,21,22,23,25,26]. Overall, the meta-analysis showed that co-infections occurred by chance (*p* = 0.097, odds ratio (OR): 0.54, 95% CI: 0.26–1.12, *I*^2^: 94.9%). Results of individual studies showed that malaria and influenza virus co-infection occurred frequently in the study conducted in the Central African Republic during 2015–2018 [13], with less co-infection occurring in the study conducted in Cambodia during 2006–2009 [25] (Figure 9).

### 3.7. Publication Bias

**Figure 10:**
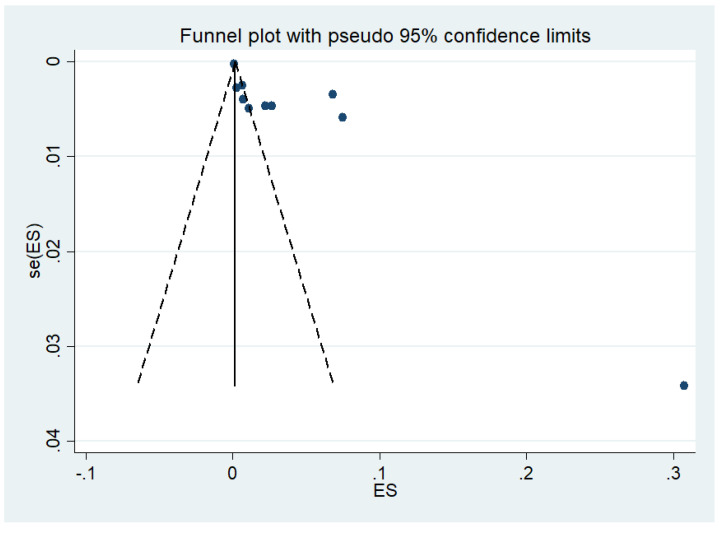
The asymmetrical distribution of the effect estimate (ES, prevalence of co-infection) and its standard error (se) is depicted in a funnel plot.

Publication bias was assessed by visualizing the funnel plot and analyzing by Egger’s test. There was an asymmetrical distribution of the studies in the funnel plot, indicating the publication bias of the prevalence of co-infection among the included studies (Figure 10). Egger’s test demonstrated that publication bias was caused by a small study effect (*p* = 0.015, co-efficient 6.97, standard error 2.26).

**Figure 11:**
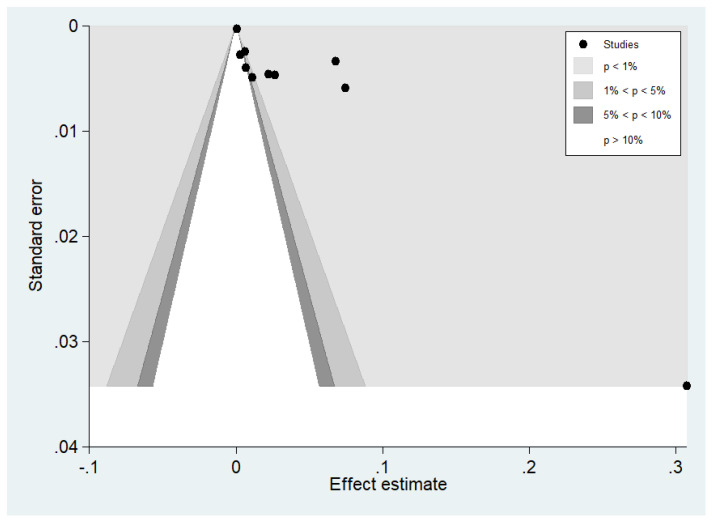
The distribution of the effect estimates of the included studies in the significant area (*p* 1%) is shown in a contour-enhanced funnel plot.

A contour-enhanced funnel plot was further created to demonstrate other possible causes of funnel-plot asymmetry. This showed that the effect estimates of the included studies were mostly located in the significant area (*p* < 1.0%), indicating that the shape of the funnel plot was likely caused by publication bias (Figure 11).

## 4. Discussion

In this study, we found that the co-occurrence of malaria and influenza infections in febrile patients were published in the literatures between 2012 and 2021. The results of the meta-analysis showed that although the overall prevalence of malaria and influenza co-infection among febrile patients was low (3.0%), the causes of high heterogeneity among studies need to be considered. The difference in the prevalence of co-infections might due to the difference in countries, participants, and diagnostic tests for influenza virus in different studies. Interestingly, influenza virus infection among malaria patients was commonly found in Africa where falciparum malaria is endemic. These findings were consistent with those that previously reported a high prevalence of influenza A virus infection among malaria patients in Lagos State, Nigeria [11]. The incidence of malaria co-infection with influenza was also reported as approximately 6.8% in the Central African Republic between 2015 and 2018 [13]. In hyperendemic areas, where malaria is endemic in both urban and rural areas, the low prevalence of malaria and influenza virus co-infection in Africa might be caused by underdiagnosis by physicians in the absence of respiratory tract symptoms of influenza at presentation or misdiagnosis of co-infection. In hypoendemic areas such as South-East Asia, malaria is frequently found in rural populations; hence, the first differential diagnosis of patients who present with fever after spending time in the forest is malaria rather than influenza. Co-infection of malaria with other AFIs was reported in our previous studies [27,28,29]. Co-infections patients were less reported, perhaps because of under-reporting or underevaluation or misdiagnosis. Nevertheless, co-infection of malaria with leptospirosis or chikungunya tended to occur by chance [27,28]. Regarding malaria and influenza co-infection, the high prevalence of co-infection under poor living conditions might be associated with poor outcomes from influenza infection, especially in developing countries [30,31]. Given that several studies from Africa have reported a high prevalence of influenza virus infection associated with hospitalization [32,33], the relevant infection control strategies, including vaccination, warrant closer attention [32].

Subgroup analysis of study populations showed that the pooled prevalence of co-infection with influenza virus infection among malaria patients was highest in all age groups (4.0%) and children (8.0%). Lower prevalence was reported by studies that enrolled specific groups, such as pregnant women and adults. These results indicated that the co-infection of these two diseases could occur in all age groups, especially in children with influenza virus infection. This result was consistent with a report on the risk and severity of co-infection among children in Kenya [14,34], which showed that 45.0% of children < 5 years old with influenza virus infections were co-infected with malaria, while only 6.0% of malaria-positive patients were co-infected with influenza [14]. In addition, children aged 5–10 years (11.0%) were co-infected with malaria and influenza [34]. Longer hospitalization of children < 5 years old for co-infection with malaria and influenza was uncommon [14]. However, two studies [11,22] that recruited pregnant patients reported a low prevalence of co-infection in this group. Therefore, pregnant women who are infected by these two pathogens might not be the source of heterogeneity in the prevalence of co-infection in febrile illness. In Nigeria, pregnant women (56.6%) were IgM seropositive for influenza A virus and co-infection with malaria (54.0%) and typhoid fever (33.0%) [11]. Moreover, the most affected patients with co-infection had the highest seroprevalence estimated to occur among adults aged 21–30 years [11]. Possibly they were active working people and had risk to contact with other people including people with influenza.

Malaria and influenza share similar clinical symptoms with other febrile illnesses at the early stages of infection, which may leads to misdiagnosis and delays optimal treatment [35]. Influenza may be undiagnosed in febrile malaria patients if clinicians do not suspect influenza co-infection. In this study, the prevalence of co-infection was stratified by diagnostic tests for influenza virus infections, including ELISA (IgM), ELISA (IgM/IgG)/PCR, and PCR. The highest prevalence of co-infection and influenza virus infection among malaria patients was diagnosed using ELISA (IgM) and PCR. Moreover, the diagnostic tool for influenza diagnosis in the study in Nigeria was ELISA. Seropositivity by ELISA is used to detect IgM-specific antibodies to influenza A virus H1N1 and H3N2 [11]. The lowest prevalence of co-infection and chikungunya infection in malaria patients was diagnosed using ELISA (IgM/IgG)/PCR. In addition, two studies [13,22] did not specify the diagnostic method for the influenza virus. Currently there are several methods for the diagnosis of influenza infections, such as real-time reverse transcription-PCR assays, viral isolation in cell culture, immunofluorescence assays, and immunochromatography assays [36].

The meta-analysis showed that malaria and influenza virus co-infection occurred by chance. The rationale behind this occurrence might be the difference in vectors responsible for transmitting the diseases. While malaria is transmitted by female *Anopheles* mosquitoes, influenza is transmitted via direct contact with infected individuals or by inhalation of virus-laden aerosols. Nevertheless, a high probability of co-infection was demonstrated in the studies conducted in the Central African Republic during 2015–2018 [13]. The results of this study indicated that two diseases might enhance another infection. However, the low probability of co-infection was reported in the study conducted in Cambodia during 2006–2009 [25], which indicated that one infection might suppress another infection. Further studies are needed to investigate the interaction between these two diseases.

The present study had some limitations. First, the pooled prevalence of malaria and influenza virus co-infection in febrile patients or the pooled prevalence of influenza virus infection in malaria patients was heterogeneous. Therefore, the pooled prevalence must be interpreted with caution. Second, the number of publications that reported malaria and influenza co-infection was limited; hence, in the present study, the differences in clinical characteristics, laboratory data, and treatment outcome of co-infected patients could not be analyzed. Third, the prevalence of malaria and influenza virus co-infection was dependent on the diagnostic tests used for the influenza virus infection, which are not all confirmatory; therefore, the rate of co-infection might have been underestimated? in some of the included studies.

## 5. Conclusions

Prevalence of malaria and influenza virus co-infection among febrile patients was heterogeneous by country, characteristics of febrile participants, and diagnostic tests for influenza virus. Clinicians examining febrile patients in co-endemic areas such as Nigeria, Tanzania, Uganda, Malawi, Ghana, Cambodia, the Central African Republic, and Kenya should carefully examine patients for the possibility of co-infection. Influenza should also be suspected in febrile malaria patients in any country during influenza season. Moreover, further studies should investigate severe clinical manifestations or differentiate clinical outcomes between mono-infected or co-infected individuals, if the co-infection leads to severe disease outcomes.

## Figures and Tables

**Table 1:** Characteristics of the included studies.

Authors, Year	Study Site	Study Duration	Study Design	Participants	Age (Years)	Age Range (Years)	Co-Infections	Malaria Cases	Test for Malaria	Test for Influenza
Anjorin et al., 2020	Nigeria	2016–2018	Observational study	182 pregnant women with influenza-like illness	Median 29	18–45	56	56	NS	ELISA IgM
Chipwaza et al., 2014	Tanzania	2013	Observational study	364 febrile children	NS	2–13	1	83	Microscopy	ELISA IgM, IgG, PCR
Cummings et al., 2021	Uganda	2017–2019	Prospective cohort study	431 febrile adults hospitalized with suspected sepsis	Median 32	IQR: 26–42	3	58	RDT	PCR
Divala et al., 2016	Malawi	2009–2010	Prospective cohort study	450 pregnant women	20.2 ± 0.25	≥15	5	88	Microscopy	NS
Hercik et al., 2017	Tanzania	2014–2015	Prospective observational study	997 febrile patients	Median 23	1–79	22	327	RDT, Microscopy	PCR
Hogan et al., 2017	Ghana	2014–2015	Prospective observational study	1063 febrile children	Median 2	IQR: 1–4	7	NS	Microscopy	PCR
Kasper et al., 2012	Cambodia	2006–2009	Prospective observational study	9997 febrile patients	Median 13	IQR: 6–28	8	716	Microscopy	PCR
Mueller et al., 2014	Cambodia	2008–2010	Prospective observational study	1193 febrile patients	Mean 23.4 ± 10.6	7–49	32	644	RDT, PCR	PCR
Nzoumbou-Boko et al., 2020	The Central African Republic	2015–2018	Retrospective observational study	5397 febrile patients	Median 11	2 months to 78 years	367	3609	RDT	NS
Thompson et al., 2012	Kenya	2009–2011	Retrospective observational study	1992 febrile patients	Mean 2.31 ± 1.34	0–5	149	1322	Microscopy	PCR

## Data Availability

All data related to the present study are available in this manuscript and supplementary files.

## Supplementary Materials

The following supporting information can be downloaded at: https://www.mdpi.com/article/10.3390/tropicalmed7080168/s1; Table S1: Search term; Table S2: Quality of the included studies.

## Abbreviations

AFIs: acute febrile illnesses; ELISA: enzyme-linked immunosorbent assay; JBI: Joanna Briggs Institute; MeSH: Medical Subject Heading; OR: odds ratio; WHO: World Health Organization.
