# Supplementary material for: Prevalence and Characteristics of Malaria and Influenza Co-Infection in Febrile Patients: A Systematic Review and Meta-Analysis
Source: Trop Med Infect Dis. 2022 Aug 5;7(8):168. doi: 10.3390/tropicalmed7080168 (PMC9413030; doi:10.3390/tropicalmed7080168)
Supplement: Supplementary file 1 [file tropicalmed-07-00168-s001.zip › tropicalmed-1827816-supplementary.pdf]

Tropical Medicine and Infectious Disease

Systematic Review

Prevalence and Characteristics of Malaria and Influenza Co-Infection in Febrile Patients: A Systematic Review and Meta-Analysis

Polrat Wilairatana [1†], Wanida Mala [2†], Kwuntida Uthaisar Kotepui [2] and Manas Kotepui [2,*]

[1] Department of Clinical Tropical Medicine, Faculty of Tropical Medicine, Mahidol University, Bangkok 10400, Thailand; polrat.wil@mahidol.ac.th
[2] Medical Technology, School of Allied Health Sciences, Walailak University, Tha Sala, Nakhon Si Thammarat 80160, Thailand; wanida.ma@wu.ac.th (W.M.); kwuntida.ut@wu.ac.th (K.U.K.)
† Equally distributed as first authors
* Correspondence: manas.ko@wu.ac.th; Tel.: +66-954392469

Table S1. Search term.

Databases	Search terms/Search strategy	Date
MEDLINE	(malaria OR plasmodium OR Paludism OR "Marsh Fever" OR "Remittent Fever") AND (influenza OR flu OR Influenzas OR Grippe)<br>Search results: 893	27 June 2021
Scopus	(malaria OR plasmodium OR Paludism OR "Marsh Fever" OR "Remittent Fever") AND (influenza OR flu OR Influenzas OR Grippe)<br>Search option: Title, abstract, keywords<br>Search results: 2459	27 June 2021
Web of Science	(malaria OR plasmodium OR Paludism OR "Marsh Fever" OR "Remittent Fever") AND (influenza OR flu OR Influenzas OR Grippe)<br>Search option: All fields<br>Search results: 901	27 June 2021

Table S2. Quality of the included studies.

No.	Authors	Eligibility criteria	Study subjects and the setting	Exposure measured in a valid and reliable way 'gold standard'	A specified diagnosis or definition	Confounding factors	Dealing with confounding factors	Outcomes measured in a valid and reliable way	Appropriate statistical analysis	Scores (8)	Risk of bias (high, moderate, low)
1	Anjorin et al., 2020	Yes	Yes	No	Yes	No	NA	Yes	Yes	6	Moderate
2	Chipwaza et al., 2014	Yes	No	Yes	Yes	No	NA	Yes	Yes	7	Low
3	Cummings et al., 2021	Yes	Yes	No	Yes	No	NA	Yes	Yes	6	Moderate
4	Divala et al., 2016	Yes	Yes	No	Yes	No	NA	Yes	Yes	6	Moderate
5	Hercik et al., 2017	Yes	Yes	Yes	Yes	No	NA	Yes	Yes	7	Low
6	Hogan et al., 2017	Yes	Yes	Yes	Yes	No	NA	Yes	Yes	7	Low
7	Kasper et al., 2012	Yes	Yes	Yes	Yes	No	NA	Yes	Yes	7	Low
8	Mueller et al., 2014	Yes	Yes	Yes	Yes	No	NA	Yes	Yes	7	Low
9	Nzoumbou-Boko et al., 2020	Yes	Yes	No	Yes	No	NA	Yes	Yes	6	Moderate
10	Thompson et al., 2012	Yes	Yes	Yes	Yes	No	NA	Yes	Yes	7	Low

NA, Not Applicable.
